# Adverse effects of Lugol’s iodine: Heart failure in a patient with subcutaneous phycomycosis from a resource-limited setting

**DOI:** 10.1016/j.mmcr.2022.09.001

**Published:** 2022-09-16

**Authors:** Dean S. Karahalios, Andrea Shaw, Bonnke Arunga, Carlee Lenehan, Valentine Sing'oei, Walter Otieno

**Affiliations:** a SUNY Upstate Medical University Department of Pediatrics, 750 East Adams Street, Syracuse, NY, 13210, USA; b SUNY Upstate Medical University Departments of Medicine, Pediatrics, and International Health, 750 East Adams Street, Syracuse, NY, 13210, USA; c Obama Children's Hospital, Jomo Kenyatta Highway Kaloleni, Kisumu, Kenya

**Keywords:** Subcutaneous phycomycosis, Fungal infection, Lugol's iodine, Heart failure, Social determinants of health

## Abstract

Subcutaneous phycomycosis becomes a chronic, debilitating condition if left untreated. Treatment includes oral antifungal therapy, though oral potassium iodide has been used in resource-limited settings. Lugol's iodine has been an effective substitute, but little is known about its safety. We report a case of subcutaneous phycomycosis complicated by heart failure during treatment with Lugol's iodine. We review subcutaneous phycomycosis, iodine-mediated cardiotoxicity, as well as social determinants of health relevant to our case, suggesting that Lugol's iodine may only be an effective treatment with proper dosing and long-term monitoring.

## Highlights

• Lugol's iodine has been an alternative therapy in resource-limited settings.
• Proper dosing and long-term monitoring is essential to prevent toxicity.
• Excess iodine can cause heart failure through hyperthyroid and hypothyroid states.
• Social determinants of health may cause dosing errors and inadequate monitoring.

## 1 Introduction

Subcutaneous phycomycosis is a rare fungal infection causing localized swelling of soft tissue first described in Indonesia in 1954 [1]. Diagnosis is clinical but is confirmed by histopathology. Treatment includes oral antifungal therapy.

In limited-resource settings where histopathology or oral antifungals may not be available or affordable, progression to severe tissue scarring and deformation occurs with associated morbidity. Morbidity results from the weight of the mass; depending on its location, it may cause an antalgic gait, inability to lift the affected extremities, abnormal posture, or restrictive breathing. Although the mass typically remains confined in the subcutaneous tissue, visceral involvement has been reported in the gastrointestinal tract and upper and lower respiratory system [2,3].

Oral potassium iodide and Lugol's iodine have been reported as successful treatments [4]. However, if iodine therapy is not administered or monitored correctly, serious morbidity can result. We describe a case of subcutaneous phycomycosis treated with Lugol's iodine and complicated by heart failure. We also discuss iodine-mediated cardiotoxicity and social determinants of health contributing to our case.

## 2 Case

An 8-year-old Kenyan male with iron deficiency anemia developed a worsening right lower extremity subcutaneous mass (day −1550, Table 1). The mass was initially located in the mid-anterior thigh and was soft with localized pruritus. There was no fever, overlying rash, or drainage. The mass grew progressively more indurated and extended from hip to foot ipsilaterally, causing dull, nonradiating pain. The mass prevented flexion of the knee and caused a limping gait such that he walked with a makeshift cane.

**Table 1:** Timeline of patient's care.

Time	Event
Day −1550	Symptom onset with swelling of right anterior thigh.
Day −1550 to day −90	Slowly progressive swelling and induration extending throughout the right leg but sparing the foot and groin. Patient was seen intermittently at local government health dispensaries and treated with benzylpenicillin, amoxicillin, and flucloxacillin without response.
Day −90	Case review by consultant pediatrician at district community hospital, where presumptive diagnosis of phycomycosis was made by history and examination. Family was not able to afford further evaluation or treatment and a limited course of itraconazole was provided from charity supply for 1 month without significant improvement.
Day 0	Referral to regional teaching and referral hospital for worsening pain that was limiting ambulation. Patient was treated for malaria and deep vein thrombosis.
Day +27	Additional limited course of itraconazole provided from charity supply for 1 month without significant improvement.
Day +48	Open biopsy confirmed subcutaneous phycomycosis.
Day +58	Patient started Lugol's iodine therapy.
Day +62	Patient able to bear weight on right leg again with reduction in swelling and increased range of motion of right knee.
Day +62 to day +97	Prolonged hospital stay involving extended time teaching about medication delivery and adherence, thyroid monitoring, and observing mother administering medication.
Day +97	Discharge home with Lugol's iodine and warfarin.
Day +104	Readmission for congestive heart failure presumably due to iodine toxicity. Lugol's iodine was discontinued and furosemide, lisinopril, and digoxin were started.
Day +142	Discharge home to continue warfarin.
Day +156	Readmission with second congestive heart failure exacerbation.
Day +172	Discharge home to continue warfarin, furosemide, lisinopril, and digoxin.
Day +248	Patient visited at home. He was no longer taking any medications as his family could not afford them and his mother was ill in hospital. Physical exam was notable for improved asymmetry of the lower extremities, but with scarring and signs of compensated congestive heart failure.

The patient lived rurally with his parents and five older siblings. His parents were subsistence farmers and his father also caught fish in a nearby lake. Neither of his parents attended formal education and they were illiterate in their native language. His family could not afford health insurance, medications, or transportation to receive regular medical care. As a result, the patient's symptoms slowly progressed for four years. During that time, he was evaluated at local government health dispensaries several times, where he was treated for presumed bacterial infection without improvement. Review of his receipts of care showed that he was treated with benzylpenicillin, amoxicillin, and flucloxacillin at various points during his course (day −1550 to day −90).

As his symptoms continued to evolve, he presented to a district community hospital for evaluation by a consultant pediatrician (day −90). Biopsy was recommended to rule out filarial disease, lymphadenitis, and malignancy, but his family could not afford it. However, the slow progression, woody enlargement, and lack of lymph node involvement made the clinical impression consistent with subcutaneous phycomycosis. A limited course of itraconazole 200 mg once daily for 30 days was donated, but a full course was not affordable or available. Despite multiple attempts to educate the patient's family, poor health literacy and lack of resources contributed to inconsistent follow up and disease progression. He presented again 3 months later with worsening pain and was transferred to a regional teaching and referral hospital where he and his mother could remain together for supervised treatment and government-subsidized care.

**Fig. 1:**
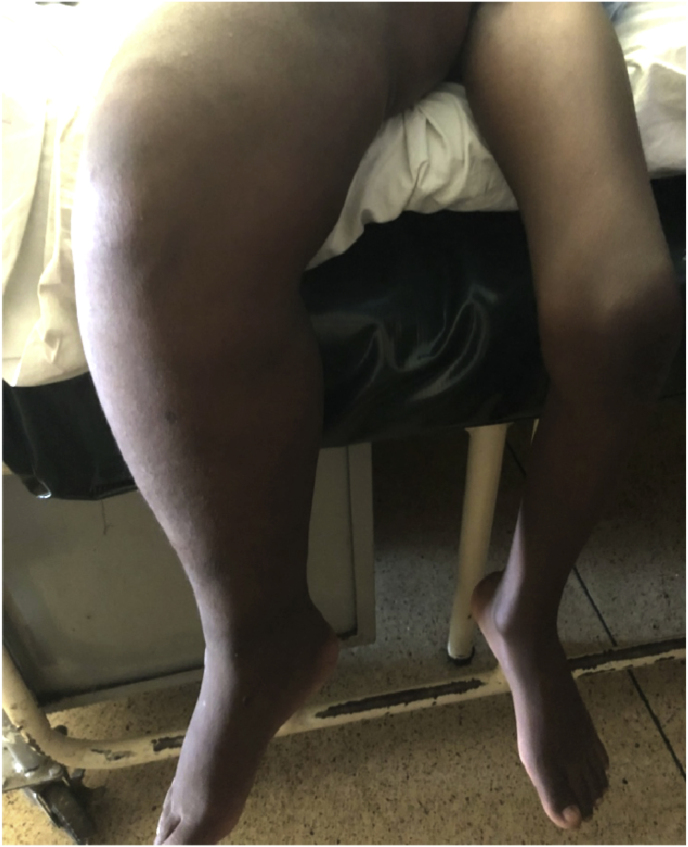
Initial presentation showing diffuse, woody enlargement of right lower extremity.

On arrival (day 0), his vital signs were normal. He complained of fatigue, headache, and heart palpitations. He was unable to bear weight on his right leg. Right mid-thigh circumference was 50cm, compared to 27cm on the left. Right tibial circumference was 22cm, compared to 13cm on the left [Fig. 1]. There was no palpable lymphadenopathy.

Laboratory evaluation revealed leukocytosis to 15,500 cells/μL and severe anemia with hemoglobin 4.5g/dL. Peripheral blood smear showed malaria infection. He was transfused with 1 unit of blood and given artemether/lumefantrine for malaria treatment. Electrolytes, blood urea nitrogen, creatinine, liver function tests, and coagulation profile were normal. Abdominal ultrasound was normal. Right lower extremity Doppler ultrasound showed deep vein thrombosis. Enoxaparin was bridged to warfarin for anticoagulation. The patient also received acetaminophen for pain and Ranferon (iron, folic acid, ascorbic acid, vitamin B12, and zinc supplementation) for anemia. His leg swelling was treated with another course of itraconazole 200 mg once daily for 30 days starting on day +27. Biopsy on day +48 revealed a polymorphous population of lymphoid cells including granulomas, neutrophils, cellular debris, and stromal fragments. There was no atypia and acid-fast staining was unremarkable. Hyphal fragments were seen, which, together with his clinical findings, confirmed the diagnosis of subcutaneous phycomycosis.

Treatment options for the patient's subcutaneous phycomycosis were limited. His family could not afford oral antifungal therapy and the medical team exhausted charity-donated supplies. Moreover, both the antifungal therapy and potassium iodide were not available locally. After reviewing literature documenting its successful treatment, Lugol's iodine was selected as an alternative. The patient was given Lugol's iodine 5 drops once daily for one day, then twice daily for one day, then three times daily thereafter starting on day +58 [4]. Before starting treatment, baseline thyroid studies were confirmed to be normal. After demonstrating a decrease in the size of his mass and improved mobility after 2 weeks of treatment in hospital (day +62), thyroid studies were repeated and were normal. His mother was educated to administer the medication daily with supervision and encouragement from the medical team. The patient was discharged on day +97 to complete a total of 3–6 months of therapy with Lugol's iodine and return for follow up with his consultant pediatrician at the district hospital in one month.

One week after discharge (day +104), the patient returned due to progressive dyspnea. Physical exam showed tachycardia, tachypnea, hypoxia, an audible S3 heart sound, bibasilar rales, jugular venous distension, hepatomegaly, and periorbital edema. Although his right lower extremity mass was smaller, he continued to have a limping gait requiring his makeshift cane. He was accompanied by his father as his mother was ill in hospital due to uncontrolled diabetes.

Laboratory evaluation revealed elevated BUN and creatinine. Abdominal ultrasound showed dilated hepatic veins. While admitted to the hospital, he was treated with furosemide, lisinopril, and digoxin for heart failure presumably due to iodine toxicity. Lugol's iodine was discontinued with improvement of his heart failure symptoms.

He was discharged on day +142 in stable condition, only to return with a second heart failure exacerbation on day +156. The patient's family restarted Lugol's iodine at home despite recommendations to discontinue therapy. After stabilization, he was discharged home to continue a low dose of lisinopril, furosemide, and digoxin on day +172. At discharge, his leg circumferences were still asymmetric, which was attributed to chronic scarring. Nevertheless, he was able to ambulate independently. Follow up after one month was recommended with his pediatrician at the district community hospital.

**Fig. 2:**
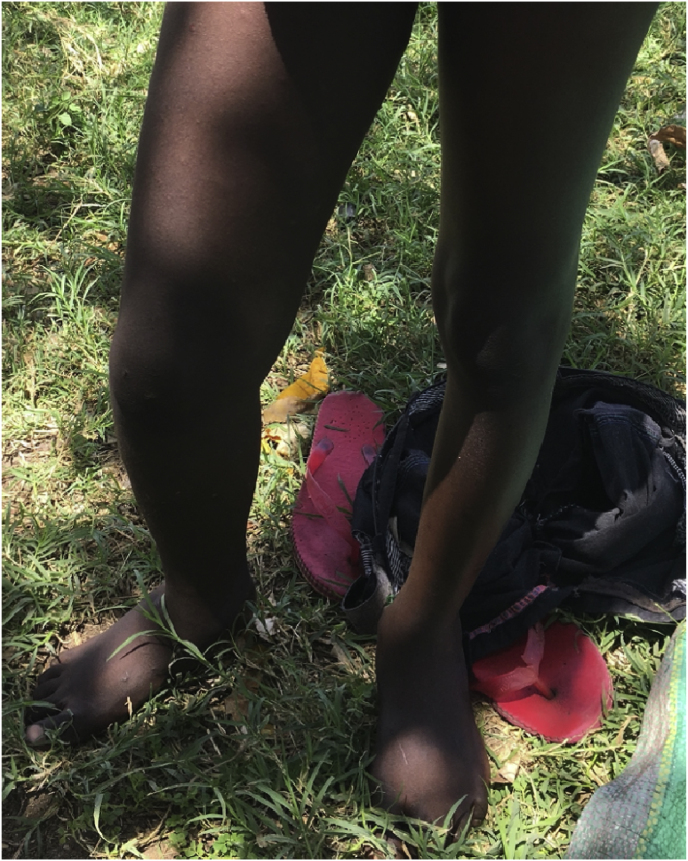
Right lower extremity following treatment.

At a home visit two months later on day +248, the patient was not taking any medications as his family was unable to afford the medications or transportation for follow up. He was mildly tachycardic with hepatomegaly. His right leg decreased in size but chronic scars remained [Fig. 2]. The right leg diameter of 32 cm was slightly larger than the left leg diameter of 27cm. Mobility was improved from the start of the treatment course, but remained reduced in the right knee.

## 3 Discussion

Subcutaneous phycomycosis is a rare fungal infection attributed to *Basidiobolus ranarum*, a fungus found in soil and in the intestines of amphibians and reptiles [5]. It thrives in warm, humid environments and thus most case reports have been reported in tropical Asia and Africa, though others have been reported in the Americas [2,5]. While 90% of cases have been reported in individuals less than 20 years old, infection can also occur in adults [1,4].

Human infection occurs through breaks in skin. The lower extremities and trunk are most commonly affected, though other involvement is possible with the proper inoculum. As seen in our patient, clinical manifestations of subcutaneous phycomycosis typically begin as a small, indurated subcutaneous mass. There are typically no overlying skin changes. The lesion is often painless, but if left untreated, can grow significantly and pain is caused by the burden of the large, woody mass [2,5]. Spread occurs along regional anatomical boundaries and disseminated infection has not been reported. Visceral involvement is uncommon given the mode of transmission, but rare cases of gastrointestinal, retroperitoneal, maxillary sinus, and lung involvement have been reported, mimicking inflammatory bowel disease, malignancy, chronic sinusitis, and lung parenchymal disease [2,3]. Diagnosis is clinical and confirmed by histopathology. KOH prep reveals wide, hyaline, thin-walled, non-septate (or infrequently septate) hyphae [5].

Some cases of subcutaneous phycomycosis are self-limiting [4]. Guidelines for management of cases that do not resolve on their own are not clear. Itraconazole is the most commonly reported effective treatment. Amphotericin B, ketoconazole, voriconazole, and posaconazole have also been reported [5]. Treatment with surgical resection alone is controversial due to risk of further spread of the infection. Many cases of subcutaneous phycomycosis treated surgically ultimately need antifungal therapy [6].

In resource-limited settings where first-line therapies are not available, oral potassium iodide has been an effective treatment at 30 mg/kg once daily or 10 mg/kg three times daily for 6–12 months [4]. In settings where even oral potassium iodide is not available, substitution with readily available Lugol's solution has been used as an alternative as it contains soluble potassium iodide. Lugol's solution is a mixture of potassium iodide 10% and iodine 5% that is antiseptic with direct antifungal properties. It is also commonly used as preparation prior to surgical thyroidectomy due to its inhibition of thyroid peroxidase, decreasing thyroid gland size and vascularity. It is commonly prescribed in a tincture and dosing is recorded in drops [7].

Thotan et al. report a case of subcutaneous phycomycosis successfully treated with a gradual increase to 15 drops of oral Lugol's iodine solution for 3 months. At 2-year follow up, the patient remained without recurrence and did not suffer any complications of treatment [4]. Nevertheless, our case should caution the future use of Lugol's iodine solution due to the inherent risk of iodine toxicity and heart failure.

Excess iodine exposure can cause heart failure due to hypothyroidism, hyperthyroidism, or direct cardiac toxicity. Acute excess iodine exposure results in transient hypothyroidism termed the Wolff-Chaikoff effect and is thought to be mediated by thyroid peroxidase inhibition. Normal thyroid hormone synthesis resumes after a period of 24–48 hours, albeit with less sodium-iodide symporter expression [8]. Chronic hypothyroidism ensues in patients who are unable to recover normal thyroid hormone synthesis following the Wolff-Chaikoff effect. Patients with history of thyroid disease such as Hashimoto's thyroiditis or subacute thyroiditis may be less likely to recover. Similarly, patients who have undergone treatment with antithyroid medication, radioactive iodine, or hemithyroidectomy are also at risk [8].

In turn, hypothyroidism itself causes heart failure through many different genetic, hormonal, and structural mechanisms. Thyroid hormone (especially T3) upregulates transcription of many important compounds implicated in cardiac contractility, such as α-myosin heavy chain and the sodium-potassium ATPase. Thyroid hormone also decreases resistance in vascular smooth muscle, leading to activation of the renin-angiotensin-aldosterone system and increasing blood volume [9]. Relative lack of thyroid hormone leads to a decrease in ventricular contractility and cardiac output through these same mechanisms. Moreover, lack of thyroid hormone leads to ventricular remodeling such that sarcomeres are lengthened and arranged in series, a phenomenon also observed in dilated cardiomyopathy [10].

On the other hand, excess iodine exposure may also cause hyperthyroidism through the Jöd-Basedow phenomenon. Hyperthyroidism is more likely to result from excess iodine exposure in patients with nontoxic goiter or thyroid nodules [8]. Excess thyroid hormone results in heart failure through the same mechanisms described above that cause an increase in resting heart rate and cardiac output. Dyspnea on exertion may develop as patients are unable to meet the demands of exercise with a heart rate already elevated at baseline. Although excess thyroid hormone initially causes ventricular hypertrophy, prolonged exposure to a high cardiac output state ultimately leads to weakening and dilation of the ventricle [10].

Considering the many adverse effects of excess iodine exposure, iodine prescription should be done with caution. To our knowledge, Thotan's case is the only one that successfully treated subcutaneous phycomycosis with Lugol's iodine. Hence, there is not enough data to support its generalizability without proper monitoring.

The dosing of Lugol's iodine in drops may also be confusing to patients. Some tinctures have markings indicating volume in milliliters, which may mislead patients to think that dosing is expressed in milliliters rather than drops [7]. This is especially an issue where access to care is limited and poor health literacy may lead to misunderstood dosing.

Indeed, it is important to acknowledge the influence of social determinants of health in our case. Limited access to care is a well-known social determinant of health that leads to poor health outcomes globally [11]. Lack of transportation and financial resources to pay for medical care contributed to the delayed diagnosis and treatment of this patient in rural Kenya. Even after diagnosis, the primary treatment for this patient's condition was not affordable and a substitute was required. Response to therapy was not closely monitored and resulted in adverse effects.

Meanwhile, low literacy and health literacy contributed to limited parental understanding of his condition, treatment rationale, careful administration, and monitoring of medications. Robbertz et al. insightfully examine the cultural issues influencing rural Kenyan mothers' and community leaders’ health literacy and response to childhood illness. Many of those interviewed properly identified illness in their children, though some, influenced by strong religious and cultural beliefs, attributed abnormal symptoms to “bewitchment.” In describing the response to childhood illness, many preferred traditional healers and chemists, saving medical professionals for severe illness. They also cite the often-confusing experience of mothers who receive advice from multiple incongruent community sources. The authors conclude that balancing medical advice and cultural norms is an important part of improving parental health literacy and health care delivery to their children [12].

The mother of the described patient was sensitively educated in her own dialect about the importance of Lugol's iodine as treatment. During her son's long inpatient admission, she was also trained to administer doses by herself. Upon returning home, her understanding of her son's condition was much improved. She unfortunately suffered from uncontrolled diabetes and was not available to provide care to him, possibly explaining his treatment complications. Moreover, his father may not have understood the medical regimen with the same clarity. Perhaps individual metabolic factors or other organic causes contributed to the differences we saw in this patient's response and in the previously reported case of subcutaneous phycomycosis treated with Lugol's iodine [4]. Regardless, where social factors impede a patient from reaching optimal health, more resources need to be invested in order to achieve equitable health outcomes.

These and other confounding factors likely apply to our case as the patient did not exhibit any adverse effects of treatment while inpatient. With that said, the importance of contextual, culturally-sensitive patient education without language barriers cannot be understated. In order to prevent errors and improve patient safety, we suggest that Lugol's iodine only be prescribed if patients' level of understanding and resources allow for proper dosing and long-term monitoring.

## Conflict of interest

There are none.

## References

[1] Joe L.K., Eng N.T., Pohan A., Van Der Meulen H., Emmons C.W. (1956). Basidiobolus ranarum as a cause of subcutaneous mycosis in Indonesia. AMA Archives of Dermatology.

[2] Gugnani H.C. (1999). A review of zygomycosis due to Basidiobolus ranarum. Eur. J. Epidemiol..

[3] Vikram H.R., Smilack J.D., Leighton J.A., Crowell M.D., De Petris G. (2012). Emergence of gastrointestinal basidiobolomycosis in the United States, with a review of worldwide cases. Clin. Infect. Dis..

[4] Thotan S.P., Kumar V., Gupta A., Mallya A., Rao S. (2009). Subcutaneous phycomycosis--Fungal infection mimicking soft tissue tumor: a case report and review of literature. J. Trop. Pediatr..

[5] Vilela R., Mendoza L. (2018). Human pathogenic entomophthorales. Clin. Microbiol. Rev..

[6] Raveenthiran V., Mangayarkarasi V., Kousalya M., Viswanathan P., Dhanalakshmi M., Anandi V. (2014). Subcutaneous entomophthoromycosis mimicking soft-tissue sarcoma in children. J. Pediatr. Surg..

[7] Sotan S., Dadwal S., Panju A. (2017). A drop or a dropperful? Overdose with an aqueous iodine oral solution (Lugol's iodine). McMaster University Medical Journal.

[8] Leung A.M., Braverman L.E. (2012). Iodine-induced thyroid dysfunction. Curr. Opin. Endocrinol. Diabetes Obes..

[9] Klein I., Danzi S. (2007). Thyroid disease and the heart. Circulation.

[10] Gerdes A.M., Iervasi G. (2010). Thyroid replacement therapy and heart failure. Circulation.

[11] Levy H., Janke A. (2016). Health literacy and access to care. J. Health Commun..

[12] Robbertz A.S., Kim S.J., Musyimi C., Tuikong S., Shanley J., Mutiso V. (2022). Qualitative analysis of health literacy: exploring a Kenyan community's response to childhood illness. Health Promot. Int..

